# Arm-in-cage testing of natural human-derived mosquito repellents

**DOI:** 10.1186/1475-2875-9-239

**Published:** 2010-08-20

**Authors:** James G Logan, Nina M Stanczyk, Ahmed Hassanali, Joshua Kemei, Antônio EG Santana, Karlos AL Ribeiro, John A Pickett, A Jennifer Mordue (Luntz)

**Affiliations:** 1 Biological Chemistry Department, Centre for Sustainable Pest and Disease Management, Rothamsted Research, Harpenden, AL5 2JQ, UK; 2 School of Biological Sciences, University of Aberdeen, Tillydrone Avenue, Aberdeen AB24 2TZ, UK; 3 Chemistry Department, Kenyatta University, P.O. Box 43844-00100, Nairobi, Kenya; 4 Behavioural and Chemical Ecology Department, International Centre for Insect Physiology and Ecology, P.O. Box 30772, GPO 00100, Nairobi, Kenya; 5 Laboratório de Ecologia Química, Departamento de Química, Universidade Federal de Alagoas 57072-970, Maceió, Alagoas, Brasil

## Abstract

**Background:**

Individual human subjects are differentially attractive to mosquitoes and other biting insects. Previous investigations have demonstrated that this can be attributed partly to enhanced production of natural repellent chemicals by those individuals that attract few mosquitoes in the laboratory. The most important compounds in this respect include three aldehydes, octanal, nonanal and decanal, and two ketones, 6-methyl-5-hepten-2-one and geranylacetone [(*E*)-6,10-dimethylundeca-5,9-dien-2-one]. In olfactometer trials, these compounds interfered with attraction of mosquitoes to a host and consequently show promise as novel mosquito repellents.

**Methods:**

To test whether these chemicals could provide protection against mosquitoes, laboratory repellency trials were carried out to test the chemicals individually at different concentrations and in different mixtures and ratios with three major disease vectors: *Anopheles gambiae*, *Culex quinquefasciatus *and *Aedes aegypti*.

**Results:**

Up to 100% repellency was achieved depending on the type of repellent compound tested, the concentration and the relative composition of the mixture. The greatest effect was observed by mixing together two compounds, 6-methyl-5-hepten-2-one and geranylacetone in a 1:1 ratio. This mixture exceeded the repellency of DEET when presented at low concentrations. The repellent effect of this mixture was maintained over several hours. Altering the ratio of these compounds significantly affected the behavioural response of the mosquitoes, providing evidence for the ability of mosquitoes to detect and respond to specific mixtures and ratios of natural repellent compounds that are associated with host location.

**Conclusion:**

The optimum mixture of 6-methyl-5-hepten-2-one and geranylacetone was a 1:1 ratio and this provided the most effective protection against all species of mosquito tested. With further improvements in formulation, selected blends of these compounds have the potential to be exploited and developed as human-derived novel repellents for personal protection.

## Background

Repellents play an important role in disrupting the interaction between mosquitoes and human beings by reducing bites [1]. Mosquito repellents are mainly accessible to people in developed countries for nuisance insects and to travellers. One of the most widely used and effective insect repellents available is the synthetic compound, *N,N*-diethyl-*m*-toluamide (DEET) and this compound is generally considered to be the "gold standard" repellent, providing long-lasting protection of up to 8 h from time of application [2]. However, there are some rare reports of severe reactions in people, additionally DEET melts plastics causing spoilage of equipment, such as glasses and mobile phones, and many consumers find the odour and sensation on the skin unpleasant [3]. For these reasons, many potential users prefer natural alternatives such as those based on plant extracts, for example, citronella oil from the *Cymbopogon nardus *plant and *p*-menthane-3,8- diol (PMD) from lemon eucalyptus (*Eucalyptus maculate citriodon*), which have good repellent properties [3-6]. The drawback of using plant-based repellents is that many of them are made up of relatively volatile constituents and are generally not effective over long periods of time and as such require frequent reapplication.

A most important use of repellents in developing countries could be a role in reducing malaria, as well as other vector-borne diseases including dengue and West Nile Virus all vectored by different mosquito species [2,3,7]. Indeed, there is recent evidence to show that repellents can significantly reduce malaria incidence by the prevention of biting of Anopheline mosquitoes by the use of topical treatments with repellents or by protection with repellent impregnated bednets [8,9]. However, most repellents are currently expensive, difficult to distribute and difficult to incorporate into local traditions and practices. The indigenous poor are not likely to purchase commercial repellent formulations and are more likely to rely on cheaper alternatives which are less effective [10,11]. In developing countries (particularly sub-Saharan Africa) alternative methods such as insecticide-treated bed nets (ITNs) and indoor residual spraying are thought to be the most appropriate method of control. However, in some regions, mosquito populations are less susceptible to these control methods because of insecticide resistance [12,13]. Additionally, alterations in behavioural patterns cause some mosquitoes to feed earlier and more frequently outdoors than previously observed [14]. This increases the contact between mosquitoes and human beings and renders ITNs less effective [11,15]. In some cases this is thought to have occurred due to selection pressure from the use of ITNs themselves. In such situations, where mosquitoes feed earlier and more readily outdoors, repellents could play a supplementary yet significant role in controlling arthropod-borne diseases [11].

Although many repellents are available, there is a need to discover a new generation of compounds that overcome the limitations of the repellents described above [1]. A repellent that is safe, cheap, and has no or little odour is desirable and this will ultimately be one which contains the lowest possible amount of active ingredients. Although a repellent for *Anopheles gambiae *mosquitoes is desirable for protection against malaria it would be highly advantageous to have one which is also effective against other mosquito species including *Aedes *and *Culex *species.

Recently, several compounds have been identified from the skin volatile profiles of human subjects that elicited little or no attractant response from *Aedes aegypti *mosquitoes in laboratory trials. These compounds are likely to be involved in the natural 'avoidance' of certain human beings by mosquitoes and, therefore, have the potential to be exploited as new, natural repellents [16]. Olfactometer experiments with *Ae. aegypti *showed that octanal, nonanal, decanal, 6-methyl-5-hepten-2-one and geranylacetone significantly 'interfered' with host-location [16]. Although it was suggested that the compounds had a repellent effect, the olfactometer used in the experiments did not allow appropriate testing of the compounds as repellents nor were field trials with volunteers a possibility at that time. However, recent field trials with *Culicoides impunctatus *midges demonstrated that 6-methyl-5-hepten-2-one, geranylacetone, decanal and octanal, on their own and in mixtures, reduced midge landings on the forearms of human volunteers when applied topically [17]. These studies suggest that the compounds could also be used as repellents against mosquitoes.

This study was designed to test the efficacy of five novel human-derived putative repellent compounds at different concentrations and in selected mixtures comprising different combinations and ratios against the malaria vector, *An. gambiae s.s*., and two other medically important mosquito species, *Culex quinquefasciatus *and *Ae. aegypti*.

## Methods

### Chemicals

6-Methyl-5-hepten-2-one (99%), nonanal (95%), decanal (95%), geranylacetone [(*E*)-6,10-dimethylundeca-5,9-dien-2-one] (96%) and DEET (97%) were obtained from Sigma-Aldrich, UK. Octanal (99%) was obtained from Fluka, UK.

### Arm-in-cage repellency trials

#### Single compounds

6-Methyl-5-hepten-2-one, octanal, nonanal, decanal and geranylacetone were tested for repellency against *An. gambiae s.s*., *Cx. quinquefasciatus *and *Ae. aegypti *mosquitoes using established methods, based on a WHO protocol [18]. *Anopheles gambiae s.s*. and *Cx. quinquefasciatus *mosquitoes were laboratory-reared at *icipe*, Kenya. *Aedes aegypti *mosquitoes were laboratory reared at UFAL, Brazil. Female mosquitoes (mated, 5-7 days post emergence) that had not previously had a blood meal and that had been starved for 18 h but previously fed on 6% glucose solution were used in experiments. Fresh cages (50 × 50 × 50 cm), with 50 female mosquitoes in each, were used for each treatment within a testing session. Six volunteers with no or little allergic reaction to bites were selected for the trials. Experiments involving *An. gambiae s.s*. and *Cx. quinquefasciatus *were done in Kenya, and experiments involving *Ae. aegypti *were done in Brazil.

Repellent compounds in acetone (ethanol for *Ae. aegypti*) solutions (0.5 ml) were applied to a volunteer's forearm from the elbow to the wrist and the hand was covered with a Nitrile glove. Acetone (or ethanol) alone (0.5 ml) served as a control on the other arm. The control arm was inserted into a cage and the number of landings was recorded over 3 min. Then the treatment arm was inserted into the same cage and the number of landings recorded in the same way. Control and treatment arms were interchanged between experimental sessions to eliminate bias.

Each compound was tested at several concentrations, starting with the lowest dose: 0.0001%, 0.001%, 0.01%, 0.1%, 1% and 10% resulting in dosages of 0.83 ng/cm^2 ^to 0.083 mg/cm^2 ^(corresponding to 500 ng to 50 mg per forearm; calculated using an estimated forearm skin area from elbow to wrist to be 600 cm^2^) [3]. Repellency data were expressed as protective efficacy (PE) and were calculated using the formula PE = (mean number of mosquitoes landed on control arm - mean number of mosquitoes landed on test arm/mean number of mosquitoes landed on control arm).

#### Unformulated and formulated mixtures

Three mixtures were tested against all three mosquito species at the same concentrations as above for single compounds and comprised: Mixture 1, 1:1:1:1:1 of 6-methyl-5-hepten-2-one:octanal:nonanal:decanal:geranylacetone, Mixture 2, 1:3:1:0.5:0.5 of 6-methyl-5-hepten-2-one:octanal:nonanal:decanal:geranylacetone (the ratios in this mixture represent the average 'unattractive' volunteer in a previous study [16], and Mixture 3, 1:1 of 6-methyl-5-hepten-2-one:geranylacetone. Due to a trend showing *Ae. aegypti *to respond more to the aldehydes than the ketones, a fourth mixture (Mixture 4) was tested with this species only. It comprised 1:1:1 octanal:nonanal:decanal.

Mixture 1 and Mixture 3 were then incorporated into a formulation which comprised emulsifying wax NF (National Formulary Emulsifying Wax), Petroleum jelly and liquid paraffin in a 1.2:2.8:1 ratio. These formulated mixtures were tested immediately after application using the above methodology and were repeated 2, 4, 6 and 8 hours after application to give PE over time for *An. gambiae s.s*., *Cx. quinquefasciatus *and every hour for 8 hours for *Ae. aegypti*. For these experiments the control comprised an arm treated with formulation only.

#### Unformulated mixtures in different ratios with *An gambiae*

The most effective repellent compounds in this study (6-methyl-5-hepten-2-one and geranylacetone) were tested together in mixtures of different ratios using the methods above at several doses (0.001%, 0.01%, 0.1%, 1% and 10%). The ratios tested were 1:0 (100%:0%), 4:1 (80%:20%), 3:1 (75%:25%), 3:2 (60%:40%), 5:4 (55%:45%), 1:1 (50%:50%), 4:5 (45%:55%), 2:3 (40%:60%) 1:3 (25%:75%), 1:4 (20%:80%) and 0:1 (0%:100%) 6-methyl-5-hepten-2-one:geranylacetone. These tests were carried out with *An. gambiae *mosquitoes only.

### Statistical analysis

Repellency data were analysed using a generalized linear model (GLM) with binomial error and logit link (logistic regression). The back-transformed treatment means were used to calculate PE and the PE standard error. The back transformed treatment means were used for treatment comparisons using least significant differences between all treatments (p < 0.05). To demonstrate synergism, a generalized linear mixed model was used to test whether the effects of the 1:1 ratio differed significantly from the sum of the effects of 1:0 plus the effects of the 0:1 ratio for each concentration. Statistical analyses were performed using GenStat Version 11 (Payne 2008).

### Ethics

This study was approved by the Grampian Research Ethics Committee (07/S0801/51). Human-bait (arm-in-cage) repellency experiments at *ICIPE *were approved by the Ethical Review Committee at the Kenya Medical Research Institute (Protocol KEMRI/RES/7/3/1).

## Results

### Arm-in-cage repellency trials

#### Single compounds/*An. gambiae*

All five human-derived compounds relating to low attractancy [16,17] and tested against *An. gambiae *gave dose-dependent repellency, with the maximum repellency observed for all compounds at 10%, the top dose tested. One hundred percent repellency was recorded for geranylacetone at 10%. DEET gave better repellency than the single compounds with 100% repellency at 1% and 10% doses (Table 1).

**Table 1:** Protective Efficacy (%) of five human-derived semiochemicals and DEET tested at different concentrations in arm-in-cage experiments

	Mean % PE (+S.E.)					
Conc. (%)	DEET	Octanal	Nonanal	Decanal	6MHO	GA
*Anopheles gambiae*						
0.0001	--	-17.4 (± 3.0)^c^*	-21.4 (± 2.9)^e^*	-13.4 (± 4.3)^e^*	0.9 (± 3.9)^c^	--
0.001	6.2 (± 1.7)^d^	-9.9 (± 1.5)^c^	-12.7 (± 2.2)^de^	5.9 (± 4.5)^de^	1.6 (± 11.2)^c^	7.3 (± 9.5)^b^
0.01	20.7 (± 6.4)^c^*	5.1 (± 5.2)^b^	-6.3 (± 2.8)^d^	9 (± 6)^d^	19.2 (± 5.5)^bc^*	11.2 (± 12.9)^b^
0.1	83.8 (± 3.9)^b^*	11.3 (± 7.2)^b^	5.7 (± 4.0)^c^	30.1 (± 7.7)^c^*	34.1 (± 6.6)^b^*	30.1 (± 11)^b^*
1	100 (± 0)^a^*	31.7 (± 5.6)^a^*	37.4 (± 5.9)^b^*	55.9 (± 6)^b^*	35.7 (± 8.7)^b^*	77 (± 5.9)^a^*
10	100 (± 0)^a^*	44.2 (± 5.4)^a^*	73.8 (± 4.7)^a^*	97.2 (± 1.4)^a^*	74.75 (± 6.1)^a^*	100 (± 0)^a^*
						
*Culex quinquefasciatus*						
0.0001	--	-47.3(± 5.1)^d^*	-45.9 (± 6.2)^e^*	-24.1 (± 8)^d^*	8 (± 1.8)^d^	4.8 (± 2.4)^d^
0.001	7.6 (± 1.8)^c^	-38.2 (± 4.1)^d^*	-27.7 (± 4.9)^d^*	-23.5 (± 4.5)^d^*	12.7 (± 2.1)^d^*	10 (± 1.6)^d^*
0.01	12 (± 1.7)^c^*	-22.8 (± 5.5)^c^*	-16 (± 3.5)^cd^*	5.7 (± 2.5)^c^	21.8 (± 4.9)^c^*	23.7 (± 4.5)^c^*
0.1	76.9 (± 5.4)^b^*	-13.5 (± 5.8)^b^*	-9.6 (± 3)^c^	37.3 (± 10)^b^*	30.1 (± 4.1)^c^*	33.2 (± 4.5)^c^*
1	98.2 (± 1.1)^a^*	-1.6 (± 3.2)^a^	23.0 (± 8.2)^b^*	91.3 (± 2.7)^a^*	49.7 (± 2.9)^b^*	84.1 (± 4.2)^b^*
10	100 (± 0)^a^*	9.8 (± 1.9)^a^	78.3 (± 2.5)^a^*	100 (± 0)^a^*	100 (± 0)^a^*	100 (± 0)^a^*
						
*Aedes aegypti*						
0.001	88.7 (± 1.9)^b^*	-11.9 (± 4.6)^c^	-21.4 (± 5.9)^c^	-18.8 (± 5.5)^d^	-27.4 (± 6.3)^d^	11.7 (± 4.5)^d^
0.01	100 (± 0)^a^*	-10.4 (± 4.3)^c^	45.5 (± 7.04)^b^	-9.4 (± 4.1)^d^	-18.9 (± 5.5)^d^	32.8 (± 6.6)^c^
0.1	100 (± 0)^a^*	24.4 (± 6.1)^b^*	53.6 (± 7.1)^b^	18.9 (± 5.5)^c^	12.3 (± 4.6)^c^	41.1 (± 6.9)^bc^
1	100 (± 0)^a^*	31.9 (± 6.6)^b^*	68.8 (± 6.6)^b^	53 (± 7.1)^b^	39.6 (± 6.9)^b^	46.7 (± 7.05)^b^
10	100 (± 0)^a^*	53.3 (± 7.1)^a^*	89.3 (± 4.37)^a^	90 (± 4.3)^a^	71.7 (± 6.4)^a^	73.3 (± 6.25)^a^

6MHO 6-methyl-5-hepten-2-one

GA geranylacetone

Means followed by different letters are significantly different from each other (P < 0.05)

P.E. = protective efficacy (% control mean - % test mean/% control mean)

*Significantly different from the control (untreated arm) (P < 0.05)

#### Single compounds/*Cx. quinquefasciatus*

For *Cx. quinquefasciatus *dose-dependent repellency was also observed, with the greatest repellency for each compound at 10%. Unexpectedly, octanal and nonanal had a strong attractive effect at low concentrations, and gave some repellency at high concentrations. Decanal, 6MHO, geranylacetone and DEET gave 100% repellency but only at a concentration of 10%. Decanal alone gave the greatest repellency (91%) at the lower concentration of 1% (Table 1).

#### Single compounds/*Ae. aegypti*

Dose-dependent repellency was observed for *Ae. aegypti *and, for each compound, the greatest repellency was achieved with the 10% concentration. Overall, DEET gave the greatest efficacy with 100% repellency achieved at all concentrations except 0.001% which gave 88.7% repellency. With this species the most effective single compounds were nonanal and decanal. At the 10% concentration decanal and nonanal gave ~90% protection which was greater than 6-methyl-5-hepten-2-one and geranylacetone which gave ~70% repellency (Table 1).

#### Unformulated mixtures/*An. gambiae*

For *An. gambiae *the greatest repellency was given by Mixture 3, which achieved 100% repellency at 1% and 10% and by Mixture 1 and 2 which achieved 100% repellency at 10%. At the lower concentration (0.1%) 87% repellency was recorded. At the same concentration, DEET gave 83% repellency. At concentration 0.01%, Mixture 3 gave 79.4% repellency, whereas DEET at 0.01% gave only 20.7% repellency (Table 2).

**Table 2:** Protective Efficacy (%) of three mixtures of human-derived semiochemicals at different concentrations in arm-in-cage experiments

	Mean % P.E. (±S.E.)			
Concentration (%)	Mixture 1	Mixture 2	Mixture 3	Mixture 4
*Anopheles gambiae*				
0.0001	--	--	9.4 (± 2.3)^d^	--
0.001	9.2 (± 1.8)^e^	-3.5 (± 6.4)^d^	24.4 (± 5.1)^c^*	--
0.01	16.9 (± 3.5)^d^*	16.5 (± 5.1)^c^*	79.4 (± 3.7)^b^*	--
0.1	44.6 (± 3.5)^c^*	26.9 (± 3.7)^c^*	87.1 (± 3.1)^b^*	--
1	94.1 (± 1.1)^b^*	59.5 (± 3.1)^b^*	100 (± 0)^a^*	--
10	100 (± 0)^a^*	100 (± 0)^a^*	100 (± 0)^a^*	--
				
*Culex quinquefasciatus*				
0.001	9.6 (± 3.5)^d^	--	11.3 (± 1.9)^d^*	--
0.01	20.82 (± 4.9)^cd^*	--	20.8 (± 2.3)^d^*	--
0.1	33.46 (± 3.8)^c^*	--	55.6 (± 5.8)^c^*	--
1	84.1 (± 7)^b^*	--	77 (± 4.5)^b^*	--
10	100 (± 0)^a^*	--	100 (± 0)^a^*	--
				
*Aedes aegypti*				
0.001	-4.5 (± 8.3)^d^	20 (± 8.3)^b^*	17.7 (± 8.3)^c^*	10.6 (± 8.3)^c^*
0.01	6.4 (± 4.9)^c^	25 (± 8.7)^b^*	8.8 (± 5.7)^c^	16.3 (± 7.4)^c^*
0.1	19 (± 7.9)^b^*	25.8 (± 8.8)^b^*	26.5 (± 8.8)^c^*	12.7 (± 6.7)^c^*
1	30 (± 9.2)^a^*	40.8 (± 9.8)^a^*	48.7 (± 9.9)^b^*	29.1 (± 9.1)^b^*
10	37.3 (± 9.7)^a^*	44.1 (± 9.9)^a^*	77.9 (± 8.3)^a^*	53.6 (± 9.9)^a^*

Mixture 1 = 1:1:1:1:1 6-methyl-5-hepten-2-one:octanal:nonanal:decanal:geranylacetone

Mixture 2 = 1:3:1:0.5:0.5 6-methyl-5-hepten-2-one:octanal:nonanal:decanal:geranylacetone

Mixture 3 = 1:1 6-methyl-5-hepten-2-one:geranylacetone

Mixture 4 = 1:1:1 octanal: nonanal: decanal

Means followed by different letters are significantly different from each other (P < 0.05)

P.E. = protective efficacy (% control mean - % test mean/% control mean)

*Significantly different from the control (untreated arm) (P < 0.05)

#### Unformulated mixtures/*Cx. quinquefasciatus*

Mixture 1 and Mixture 3 gave 100% repellency at 10% for *Cx. quinquefasciatus*. At lower concentrations, Mixture 1 gave greater repellency than Mixture 3 with 84% and 77% repellency achieved respectively at a concentration of 1% (Table 2).

#### Unformulated mixtures/*Ae. aegypti*

All of the mixtures tested against *Ae. aegypti *gave dose-dependent repellency, with the highest repellency for each mixture observed at 10%. Mixture 3 gave the greatest repellency overall (78%) at the 10% concentration. Mixture 1 gave significant repellency at 0.1%, 1% and 10% doses, but the greatest repellency (37.3%) was at a concentration of 10%. Mixture 1 gave significant a maximum repellency of 37.3%; Mixture 2, which was prepared to represent an unattractive human individual in a ratio of 1:3:1:0.5:0.5, gave 44.1% repellency and Mixture 4 achieved 53.6% repellency, all at a concentrations of 10%.

#### Formulated mixtures/*An. gambiae*

For *An. gambiae *both formulated mixtures gave 100% repellency at the start of the experiments (i.e. time zero). Around 90% repellency was achieved after 2 hours with Mixture 1 and this decreased over time to give 12% efficacy after 8 hours, which was not significantly different from the formulation control. Greater repellency was observed for Mixture 3 with 98% repellency recorded after 2 hours. Repellency then decreased to 64% after 4 hours and to 35% after 8 hours. All time periods, with the exception of 8 hours for Mixture 1, were significantly different from the formulation control. DEET gave the best repellency by maintaining 100% protection for up to 6 hours and 94% after 8 hours (Table 3).

**Table 3:** Protective Efficacy (%) of two formulated mixtures of human-derived semiochemicals and DEET (10% concentration) in arm-in-cage experiments

Time post application (hrs)	DEET	Mixture 1	Mixture 3
*Anopheles gambiae*			
0	100 (± 0)^a^*	100 (± 0)^a^*	100 (± 0)^a^*
2	100 (± 0)^a^*	89.4(± 3.7)^a^*	98.5 (± 1.51)^a^*
4	100 (± 0)^a^*	54.9 (± 4.5)^b^*	64.3 (± 6.15)^b^*
6	100 (± 0)^a^*	32.1 (± 5.6)^c^*	38.8 (± 4.31)^c^*
8	94.4 (± 1)^b^*	12.5 (± 2.1)^d^	30.8 (± 6.96)^c^*
			
*Culex quinquefasciatus*			
0	100 (± 0)^a^*	100 (± 0)^a^*	100 (± 0)^a^*
2	100 (± 0)^a^*	89 (± 4.2)^a^*	98.6 (± 1.43)^a^*
4	100 (± 0)^a^*	56.2 (± 4.4)^b^*	80 (± 1.7)^b^*
6	100 (± 0)^a^*	28.5 (± 5.2)^c^*	60.9 (± 3.1)^c^*
8	93.3 (± 4.08)^b^*	19.8 (± 3.2)^c^*	45.8 (± 4.1)^d^*
			
*Aedes aegypti*			
0	100 (± 0)^a^*	46.5 (± 9.9)^a^*	60 (± 9.8)^a^*
1	100 (± 0)^a^*	46.1 (± 9.9)^a^*	43.6 (± 9.9)^ab^*
2	100 (± 0)^a^*	13.8 (± 6.9)^b^*	30.9 (± 9.2)^bc^*
3	100 (± 0)^a^*	-8.6 (± 5.6)^b^	21.1 (± 5.6)^cd^*
4	100 (± 0)^a^*	17.2 (± 7.6)^bc^	11.3 (± 6.3)^d^
5	100 (± 0)^a^*	10.3 (± 6.09)^bc^	7.04 (± 5.1)^d^
6	100 (± 0)^a^*	-15.5 (± 7.24)^c^	-16.9 (± 7.2)^e^
7	97 (± 1.95)^b^*	1.7 (± 2.6)^c^	-2.8 (± 2.6)^e^
8	96.1 (± 1.94)^b^*	-8.6 (± 5.6)^c^	-2.9 (± 3.3)^e^

Mixture 1 = 1:1:1:1:1 6-methyl-5-hepten-2-one:octanal:nonanal:decanal:geranylacetone

Mixture 3 = 1:1 6-methyl-5-hepten-2-one:geranylacetone

Means followed by different letters are significantly different from each other (P < 0.05)

P.E. = protective efficacy (% formulation control mean - % test mean/% formulation control mean)

*Significantly different from the control (formulation-treated arm) (P < 0.05)

#### Formulated mixtures/*Cx. quinquefasciatus*

With *Cx. quinquefasciatus *both formulated mixtures gave 100% repellency at the start of the experiment, and this decreased slightly to 89% for Mixture 1 and to 99% for Mixture 3 after 2 hours. After 8 hours, the repellency given by Mixture 1 had decreased to 20%. However, for Mixture 3, repellency was maintained at a greater level, decreasing only to 80% after 4 hours, 60% after 6 hours and 45% after 8 hours. DEET gave 100% repellency up until 6 hours and this decreased to 93% after 8 hours (Table 3).

#### Formulated mixtures/*Ae. aegypti*

For *Ae. aegypti *the formulated Mixture 1 gave around 45% repellency at the start of the experiment and maintained this repellency for 1 hour. It then decreased rapidly to 13% after 2 hours and beyond this time gave no significant protection. Mixture 3 gave greater repellency (60%) at the start of the experiment. This reduced to 43.6% after 1 hour, 30% after 2 hours and 21% after 3 hours. Beyond this time, no significant repellency was achieved (Table 3).

#### Unformulated mixtures in different ratios with *Ae. aegypti*

**Figure 1:**
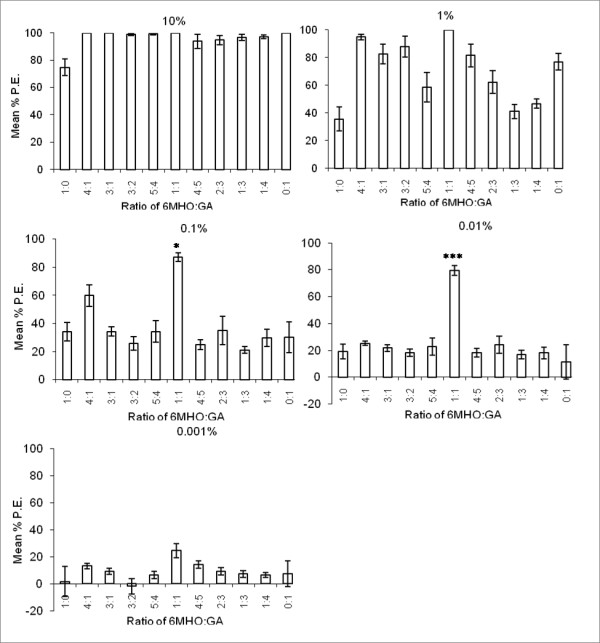
**Protective efficacy (% PE) of several mixtures of 6-methyl-5-hepten-2-one and geranylacetone in different ratios and tested at different concentrations in arm-in-cage experiments with *An. gambiae *mosquitoes**. Asterix indicate significant differences between 6-methyl-5-hepten-2-one (6MHO) alone and Mixture 3 (1:1, 6MHO:GA) and between geranylacetone (GA) alone and Mixture 3 (1:1, 6MHO:GA) (*p < 0.05; ** p < 0.01; ***p < 0.001).

All ratios of Mixture 3 (6-methyl-5-hepten-2-one with geranylacetone) provided significant repellency at 10%. However, the mixtures that contained ratios with relatively more 6-methyl-5-hepten-2-one than geranylacetone were more effective (achieving between 99% and 100% repellency) than those that contained more geranylacetone than 6-methyl-5-hepten-2-one (achieving between 93% and 97% repellency) (Figure 1). This effect was more marked at the intermediate concentrations. For two concentrations, 0.01% and 0.1%, the 1:1 ratio was more effective than the sum of effects of individual ratios 0:1 and 1:0, with statistical significance p = 0.0257 and p < 0.0001, respectively. At the lower doses, there appeared to be little difference between the different ratios although the 1:1 mixture gave the greatest protection which was significantly greater than the two compounds on their own (Figure 1).

## Discussion

### Single compounds

The three aldehydes tested in this study provided varying degrees of protection. Decanal provided the greatest repellency at the highest concentration (10%) for all three mosquito species tested (Table 1). Similarly, Logan et al. [16] demonstrated that decanal reduced *Ae. aegypti *flight activity to levels equal to a clean air control - significantly more than octanal and nonanal. Octanal provided significant repellency but only for *Ae. aegypti *and *An. gambiae*. Douglas et al. [19,20] also demonstrated that octanal was repellent against *Ae. aegypti *mosquitoes. In their study they identified octanal (and other aldehydes) from seabird odour and suggested that aldehydes are produced by birds as natural protection against ectoparasites [20]. Whilst this might be true for some arthropods, *Ae. aegypti *tend to feed on mammals (mainly humans in urban areas) and would not normally be attracted to birds. Some *Culex *species, including *Cx. quinquefasciatus*, are known to be ornithophilic and so it might be expected that their response to the aldehydes would be different from that of *Ae. aegypti*. When octanal was tested against *Cx. quinquefasciatus*, it provided no protection at all from biting. In fact, the presence of octanal (and the other aldehydes), particularly at the low concentrations, significantly increased the attractiveness of the volunteer's arms during the tests. Indeed other studies have demonstrated that nonanal elicits electrophysiological and "attractive" behavioural responses in *Cx. quinquefasciatus *mosquitoes [21-23]. Although one study demonstrated that *Ae. aegypti *are attracted to odour from bird feathers [24], *Ae. aegypti *and *An. gambiae *are normally anthropophilic and, therefore, the aldehydes tested in this study could form part of a kairomonal avian blend for bird-feeding mosquitoes, but may signal that the host is 'inappropriate' for non-bird feeding insects like *Ae. aegypti *and *An. gambiae *mosquitoes. The aldehydes may hold promise as attractants which could be used in traps (particularly for *Cx. quinquefasciatus*), although this requires further investigation. As a topically applied repellent, the lower homologue and more volatile octanal is not a good candidate, however, nonanal and decanal at high concentrations show somewhat greater promise. But these compounds were tested over a short time period only.

All three mosquito species responded to the two ketones in a dose-dependent manner and both compounds gave significant protection at high concentrations (this was particularly the case for 6-methyl-5-hepten-2-one). Similarly, Logan *et al *[16] showed that these compounds significantly affected *Ae. aegypti *flight activity and relative attraction in an olfactometer. Both compounds have also been shown to reduce the attraction of *An. gambiae *in response to a mixture of ammonia, lactic acid and carboxylic acids in an olfactometer [25]. In the field, 6-methyl-5-hepten-2-one is also known to reduce the numbers of the cattle flies, *Musca autumnalis *(Diptera: Muscidae) and *Haematobia irritans *(Diptera: Muscidae) landing on cattle [26]. Although geranylacetone gave the greatest repellency overall for two of the mosquito species tested, 100% repellency was only achieved with the 10% dose.

### Unformulated mixtures and ratios

When all five compounds were combined into a 1:1:1:1:1 ratio (Mixture 1), 100% repellency was achieved with the 10% dose with *An. gambiae *and *Cx. quinquefasciatus *(37.3% for *Ae. aegypti *at 10%), but there did not appear to be any greater effect of this combination compared with the single compounds (Table 2). Similarly, when the compounds were tested against *An. gambiae *in a mixture (1:3:1:0.5:0.5, Mixture 2), which was designed to replicate the ratios produced naturally by a relatively "unattractive" human [16] there was very little additive effect. In fact, this ratio gave lower protection than Mixture 1. However, when the aldehydes were removed to give Mixture 3 (1:1 6-Methyl-5-hepten-2-one:geranylacetone), a striking effect was observed, particularly at low concentrations. For example, with *An. gambiae *87% repellency was recorded for the 0.1% dose that exceeded (although not significantly) DEET at the same concentration, which gave 83% repellency. At an even lower concentration (0.01%), Mixture 3 maintained a high level of repellency (80%), whereas DEET gave only 20% repellency. This was a clear demonstration of synergism, as the mixture provided better protection than the sum of the repellency achieved by the single compounds at the same concentration. For example, at the 0.1% dose for *An. gambiae*, 6-methyl-5-hepten-2-one alone gave 34.1%, geranylacetone alone gave 30.1% repellency and Mixture 3 gave 87.1% repellency. At the 0.01% dose, 6-methyl-5-hepten-2-one alone gave 19.2%, geranylacetone alone gave 11.2% repellency and Mixture 3 gave 79.4% repellency. This is the first demonstration whereby adding two natural, host-derived compounds together can create a synergistic effect to produce a more potent repellent (as shown in Figure 1). Because of this result, the relative amounts of each compound in Mixture 3 were altered to determine whether a different ratio would provide better protection against *An. gambiae *than Mixture 3 (1:1 ratio).

As expected all ratios of Mixture 3 provided excellent protection at 10%, however, the effect of altering the ratios was apparent at the lower concentrations. The mixtures containing a relatively greater proportion of 6-methyl-5-hepten-2-one than geranylacetone were more effective than those that contained a greater proportion of geranylacetone than 6-methyl-5-hepten-2-one (Figure 1). In the Logan *et al*. study [16], these compounds were found to occur in "unattractive" people on average in a 1:0.7 (6-methyl-5-hepten-2-one:geranylacetone) ratio and in "attractive" people in a ratio of 1:2 [16]. Therefore, the 1:1 ratio tested in this study (Mixture 3) more closely resembled the ratio found in an "unattractive" individual than in an "attractive" person. At the lower doses, there appeared to be little difference between the ratios, although Mixture 3 (1:1) always gave the greatest protection overall and thus shows promise as a repellent for all three species tested in this study.

### Formulated mixtures

For most repellents that contain volatile compounds a slow release formulation is required to provide controlled release over time. The loss of efficacy of repellents over time is usually due to evaporative loss, dermal absorption, and abrasive loss or through the effects of perspiration and washing [1,27,28]. In this study, a basic wax-formulation was used to provide slow release of compounds. For all species, repellency decreased over time, but for *An. gambiae *and *Cx. quinquefasciatus *a high level of repellency was maintained up to 2-4 hours (Table 3). Although 6-methyl-5-hepten-2-one and geranylacetone seem to have a potent effect on mosquitoes (as demonstrated by the large level of protection provided by low concentrations) their efficacy is not long lived and does not last as long as DEET, which can remain effective for up to 8 hours. Recently, Moore *et al *[11] demonstrated efficacy of a low-cost repellent containing PMD and lemongrass oil (with fixatives) for up to 6 hours, which was more effective than DEET, against *Anopheles darlingi *in Peru. They suggest this would be a suitable repellent for malaria-endemic countries. However, even compounds like DEET and PMD, that have low volatilities in comparison to the compounds in this study, are only effective over time at or above doses of 10%. Due to the high volatility of the compounds in this study they evaporated from the skin more quickly than DEET. New advances in formulation technologies could be exploited to develop appropriate formulations that achieve slower release rates over time and this should result in the volatile compounds achieving efficacy over time that matches or exceeds that of DEET. A period of longer than 6-8 hours protection is desired. Further work is underway to develop a formulation for topically-applied preparations using these human-derived repellents.

The two ketones tested here have the potential to be used as active ingredients in new topical repellent formulations. In initial studies, the chemicals had a significant repellent effect in small quantities which suggests that, providing a suitable formulation is developed, a repellent could be created that is both odourless with little or no irritation to the skin and with no untoward side effects - characteristics that are consumer pleasing, yet not met by the current repellents on the market [29]. Additionally, 6-methyl-5-hepten-2-one and geranylacetone are acceptable food additives and are, therefore, unlikely to cause any toxicological complications (JEFCA website, 2005). The compounds could also be impregnated into clothing or onto bednets, the latter of which could be combined with insecticide treatments. However, to be effective and economically viable (especially in developing countries), these chemicals must be obtained with relative ease and low cost.

Plants often offer alternative cheap and renewable resources for semiochemical production, especially in resource-poor afflicted countries. This has been demonstrated for the *Cx. quinquefasciatus *oviposition pheromone (5R,6S)-6-acetoxy-5-hexadecanolide, where the precursor (Z)-5-hexadecenoic acid can be obtained from seed oil of a renewable plant resource, *Kochia scoparia *(Chenopodiaceae) [30-33]. Using this method, the pheromone can be obtained at a cost of $3 per gram which compares favourably with conventional synthetic methods which cost $15 per gram [31]. Similarly, the sandfly pheromone, (S)-9-methylgermacrene-B, can be synthesized from germacrone, a major component of *Geranium macrorrhizum *(Geraniaceae) essential oil [34]. Since 6-methyl-5-hepten-2-one and geranylacetone are commonly produced by plants a similar process could be used to obtain the compounds in this study and further work is underway to discover suitable plant sources of the two monoterpenes [35].

## Conclusion

Two compounds, 6-methyl-5-hepten-2-one and geranylacetone, when combined in a 1:1 mixture show promise as a topical repellent that is effective over a short time period. Further work with formulation technologies that will allow a slower release of the compounds is ongoing and should provide a repellent that is effective over several hours. Although the high volatility is a disadvantage when developing a topical repellent, it could be advantageous if the compounds affect mosquito behaviour some distance from the host. Indeed, the compounds identified here have already been shown to affect flight activity and attraction towards host odour in an olfactometer [16]. A "spatial repellent", released from a dispenser, would be a major advantage and could result in the development of a new class of mosquito repellents that provide protection within a given area without the need for topical application. Further work is underway to assess 6-methyl-5-hepten-2-one and geranylacetone as spatial repellents.

## Competing interests

The authors declare that they have no competing interests.

## Authors' contributions

JGL, AJM, AH and JAP contributed to design of the study, conceived the protocol and provided funding. The study was coordinated by JGL. NS and JGL performed the data analysis and interpretation. NS, KR, JGL, AH contributed in the study design and in the implementation of the research. JGL, AJM, NS contributed to manuscript drafting. All authors read and approved the final manuscript.
